# The IRX1/HOXA connection: insights into a novel t(4;11)- specific cancer mechanism

**DOI:** 10.18632/oncotarget.9241

**Published:** 2016-05-09

**Authors:** Alessa Kühn, Denise Löscher, Rolf Marschalek

**Affiliations:** ^1^ Institute of Pharmaceutical Biology/DCAL, Goethe-University of Frankfurt, Biocenter, D-60438 Frankfurt/Main, Germany

**Keywords:** MLL-r leukemia, HOXA profile, IRX1, HOXB4, EGR1/2/3

## Abstract

One hallmark of *MLL*-r leukemia is the highly specific gene expression signature indicative for commonly deregulated target genes. An usual read-out for this transcriptional deregulation is the *HOXA* gene cluster, where upregulated *HOXA* genes are detected in *MLL-r* AML and ALL patients. In case of t(4;11) leukemia, this simple picture becomes challenged, because these patients separate into *HOXA*^hi^- and *HOXA*^lo^-patients. *HOXA*^lo^-patients showed a reduced *HOXA* gene transcription, but instead overexpressed the homeobox gene *IRX1*. This transcriptional pattern was associated with a higher relapse rate and worse outcome. Here, we demonstrate that IRX1 binds to the MLL-AF4 complex at target gene promotors and counteract its promotor activating function. In addition, IRX1 induces transcription of *HOXB4* and *EGR* family members. *HOXB4* is usually a downstream target of c-KIT, WNT and TPO signaling pathways and necessary for maintaining and expanding in hematopoietic stem cells. EGR proteins control a p21-dependent quiescence program for hematopoietic stem cells. Both IRX1-dependend actions may help t(4;11) leukemia cells to establish a stem cell compartment. We also demonstrate that HDACi administration is functionally interfering with IRX1 and MLL-AF4, a finding which could help to improve new treatment options for t(4;11) patients.

## INTRODUCTION

*MLL*-rearrangements are associated with the onset and development of acute leukemia. So far, over 80 direct MLL fusions (MLL-X) and more than 120 reciprocal MLL fusions (X-MLL) have been described at the molecular level [1].

Experiments performed in different labs have already demonstrated that the expression of various chimeric *MLL* fusion alleles was sufficient to drive the onset of leukemia [2–8]. It is presumably the only genetic mutation required for disease onset [9]. However, also secondary mutations (e.g. mutant *RAS*) have been identified in leukemic blast cells, but their functional importance is yet not completely clear.

One reason why rearrangements of the *MLL* gene display *per se* such a profound effect is due to the crucial function of the wildtype MLL protein: MLL together with other proteins form a complex that marks promotors for gene transcription in a cell-type specific manner, thereby creating a “transcriptional memory system”. This system maintains the “lineage identity” of differentiated cells in a mitotically stable manner. It is being set by distinct histone modifications in promoter regions (MLL complex: H3K4_me3_, H3/4-Ac) and the transcribed gene body (AF4/AF5 SEC: H3K36_me2_ and H3K79_me2/3_). In addition, the MLL complex is required for embryonic as well as for adult hematopoietic stem cell maintenance [10]. By contrast, MLL fusion proteins deriving from a large variety of genetic rearrangements are strongly disturbing these fine-tuned processes. They also enable the development of tumors by establishing an “oncogenic transcriptional memory system” to maintain an aberrant transcriptional program [11–14].

Early on, MLL fusions were shown to aberrantly activate the transcription of *HOXA* genes [15,16]. All yet tested direct MLL fusions (MLL-X) activate a distinct set of *HOXA* genes, generating a distinct *HOXA* profile, which differs slightly in AML or ALL patients but always activate *HOXA9*. HOXA proteins are *per se* able to form complexes with MEIS and PBX proteins [17]. These ternary complexes are usually expressed at higher levels in stem/progenitor cells but are subsequently decreasing during final differentiation. An aberrant high expression of these proteins causes a block of normal hematopoietic differentiation and concomitantly increases the proliferation potential. An aberrant activation of *HOXA* genes is thus regarded as a “hallmark of *MLL-r* leukemia”.

However, it is not clear whether the observed *HOXA* signatures are causative or indicative for *MLL-r* leukemia. Aberrant *HOXA* gene expression - in particular *HOXA9 -* leads to an enhanced colony forming capacity [18] but the expression of MLL-AF9 in a *Hoxa9^−/−^* genetic background could not prevent leukemia development [19]. In addition, t(4;11) ALL patients can be separated into patients with and without the typical “*HOXA* signature” [20]. Nearly half of the investigated patients displayed the absence of *HOXA* transcript levels (*HOXA*^lo^-patients), but overexpressed the homeobox gene *IRX1*. The presence of *HOXA*^lo^- and *HOXA*^hi^-leukemia patients had been verified independently by a second group which correlated the high IRX1 expression to a 3-times higher risk of relapse [21]. Finally, a third group associated the missing *HOXA* signatures in t(4;11) leukemia patients with the overexpression of *IRX1/2* [22]. In addition, risk prediction of the complete *HOXA*^hi^- and *HOXA*^lo^-patient cohort was dependent on 3 key genes, namely *FLT3*, *TACC2* and *IRX2*. High expression of non-mutated *FLT3* is a typical feature in t(4;11) leukemia. However, the EFS dropped from 64 % to 15 % when *TACC2* and *IRX2* were present.

In the mouse system, the Irx1 homeobox protein is involved in embryonic patterning. It is expressed in early mesoderm (E7.5), later in the neural tube, mesencephalon and eye (E8.5). It is also strongly expressed during brain development (E10.5) and is finally involved in digit, lung, heart and kidney development (E11.5-14.5). A homozygous knock-out of *Irx1* is embryonically lethal at E9.5 and displays no gastrulation which occurs at E5.5 [23]. Importantly, Irx1 and its human counterpart IRX1 are usually not expressed in hematopoietic tissues.

Therefore, we aimed to investigate the molecular mechanisms being responsible for the phenomenon of the observed differential *HOXA* expression in t(4;11) leukemia patients and the molecular effects that are caused by the ectopic expression of IRX1. For the purpose of our study, we used an optimized gene transfer system [24] to generate stable cell culture models which allowed us to investigate the molecular consequences of induced IRX1, MLL-AF4 or the combination of both. According to our data, IRX1 expression changes the functional properties of MLL-AF4 at target gene promotors. Apart from this unique function, ectopic IRX1 expression correlates with *HOXB4* and *EGR* gene activation. The latter are well known for their importance in stem cell maintenance and expansion. This could potentially explain the increased risk of t(4;11) patients when expressing IRX1 instead of *HOXA* genes. To this end, our data provide the first rational hint for the published observations made for t(4;11) leukemia patients.

## RESULTS

### Overexpression of IRX1 revealed a complex network of target genes

**Figure 1:**
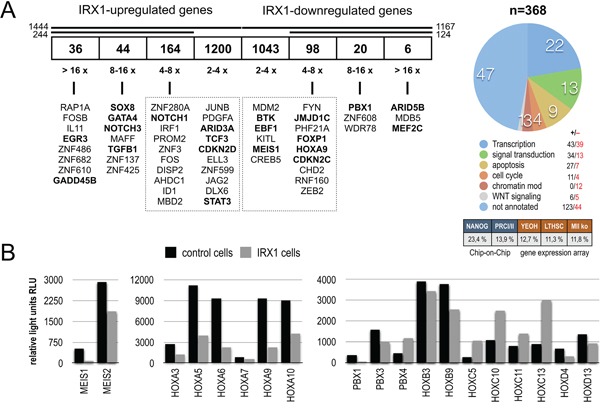
IRX1 overexpression in HEK293T cells **A.** Transiently transfected HEK293T cells expressing either IRX1::GFP or GFP were FACS-sorted. Total RNA from 2 × 10^7^ GFP-positive cells was isolated and used for hybridization on Affymetrix HG-U133Plus 2.0 microarrays (n=3). More than 8,000 genes were differentially expressed. We shortened the list of genes for experimental work on those which were deregulated more than 4-fold (up: n=244; down: n=124). All genes of this highly deregulated group were annotated according to GO terms. Nearly a quarter of genes encoded for transcription factors. The number of up- and downregulated genes is indicated. **B.** The normalized Affymetrix chip hybridization signals (relative light units RLU) are displayed for *MEIS* genes, *PBX* genes and genes of the *HOXA/B/C/D* cluster. All genes displayed were significantly deregulated.

Gene profiling experiments with HEK293T cells expressing either IRX1::GFP or GFP (mock control) were performed. Transiently transfected cells had been FACS-sorted by their GFP expression before RNA was isolated and used for Affymetrix HG-U133 Plus 2.0 chip hybridization experiments. This resulted in a data set summarized in Figure 1A (left panel). More than 8,000 genes were significantly deregulated after 48 h of IRX1 overexpression. In total, 2,611 genes were deregulated at least more than 2-fold, and 358 genes more than 4-fold. Within this group of deregulated genes, the following transcription factors - selected because of their importance for the hematopoietic system - displayed the highest upregulation: *EGR3*, several zinc finger genes, *GADD45B*, *SOX8*, *GATA4*, *NOTCH3*, *TGFB1*, *NOTCH1*, *ARID3A, TCF3*, *CDKN2D and STAT3*. By contrast, *ARID5B*, *MEF2C*, *PBX1*, *JMJD1C*, *FOXP1*, *HOXA9, CDKN2C, BTK, EBF1 and MEIS1* were the most downregulated genes. Since IRX1 expression was associated with the decrease of *HOXA* gene transcription, we displayed the relative light units to visualize the changes in gene transcription levels for *HOX*, *MEIS* and *PBX* genes (Figure 1B). Overexpression of IRX1 was sufficient to downregulate distinct *HOXA*, *HOXB* and *HOXD* genes while several *HOXC* genes were upregulated. Similarly, *MEIS1* and *MEIS2*, as well as *PBX1*, *PBX3* and *PBX4* were downregulated more than 2-fold. We also took a look to GO terms and investigated potential overlaps with other available gene expression profiles or Chip-on-Chip data sets (Figure 1A, right panel). About 22 % of all deregulated genes were transcription factors. Interestingly, the obtained signature was highly concordant with the stem cell signature that has been established for the NANOG, POU5F1/OCT4 and SOX2 which wires the network in embryonic stem cells [25]. Noteworthy, *IRX1* is a downstream target of NANOG/OCT4 and SOX2 in embryonic stem cells. However, some overlap was also evident with identified Polycomb-group target genes (13.9 %) and other published gene signatures (leukemic signature, LT-HSC and *MLL* k.o.).

Since all these data were obtained from transient transfection experiments, where the IRX1 protein was highly overexpressed for 48 h, we developed a cell culture system where IRX1 can be inducibly expressed from a vector system that is stably integrated into the genome. For this purpose, we used our recently optimized Sleeping Beauty Transposon vector system which integrates few copies of any transgene [24]. Subsequently, transgenes can be induced by the administration of doxycycline. This novel expression system displays no basal expression of transgenes but constitutively expresses a fluorescent protein and a selection marker. Since IRX1 expression turned out to be instructive, we decided to retain the HEK293T cell system in order to make our data comparable with data obtained from the previous experiments shown in Figure 1.

### IRX1 inhibits the activator function of MLL-AF4 on *HOXA* gene transcription

**Figure 2:**
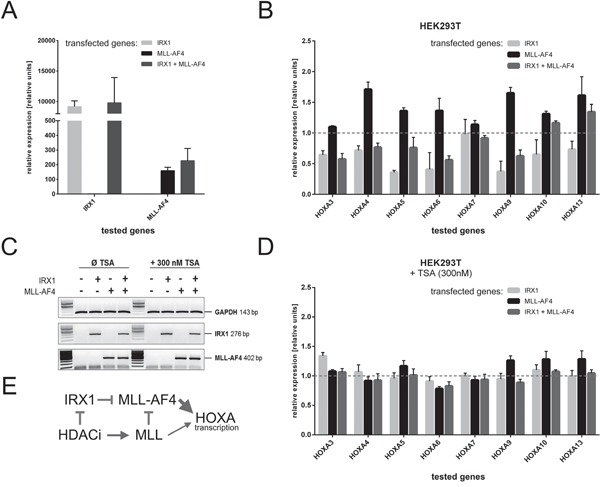
Inhibition of HOXA gene transcription in the presence of IRX1 Stable HEK293T cell lines expressing IRX1 and/or MLL-AF4 were established to investigate *HOXA* gene transcription. **A.** Transcription of all transgenes after induction with doxycyline for two days (n=3). All transgenes were transcribed as expected. **B.** *HOXA* gene transcription levels (mock-transfected cells set to 1, normalized to GAPDH) in the presence of either IRX1 (light grey), MLL-AF4 (black) or IRX1/MLL-AF4 expressing cells (dark grey)(n=3). Except for *HOXA7*, all tested *HOXA* genes were downregulated in the presence of IRX1 and upregulated upon MLL-AF4 expression. Co-expression of IRX1 and MLL-AF4 leads to a downregulation of *HOXA* gene transcription. **C.** Validation of the effect of applied TSA administration. The control gene GAPDH used for Q-PCR experiments and the expression of the two induced transgenes were not affected. **D.** Same experiment as shown in B., however, with 300 nM TSA (n=3). **E.** Simple scheme to summarize that IRX1 acts dominant over MLL-AF4, while TSA blocks the action of IRX1 and MLL-AF4 while concomitantly activating the endogenous MLL.

The *IRX1* cDNA was cloned into the doxycycline-inducible Sleeping Beauty system (*pSBtet* vectors; [24]) and stably transfected into HEK293T cells. Similarly, we established a second and a third stable cell line expressing MLL-AF4 or IRX1/MLL-AF4. As summarized in Figure 2A, we verified the proper transcription of the transgenes upon doxycycline administration in the single- or double-transfected cell lines.

Next, we studied the transgene's effects on *HOXA* gene transcription. Figure 2B displays Q-PCR data which were normalized to *GAPDH* of the mock-control cell line. With the exception of *HOXA7*, IRX1 was able to significantly decrease the transcription of the investigated *HOXA* genes. The lower expression of *HOXA3, 4, 5, 6, 9, 10* and *13* is presumably correlated with a slightly decreased transcription of endogenous MLL (~0.8-fold) in the presence of IRX1. As expected, the expression of MLL-AF4 increased the transcriptional activity of the investigated *HOXA* genes. However, when IRX1 and MLL-AF4 were co-expressed, *HOXA* gene transcription decreased again, indicating that IRX1 is acting in a dominant-negative manner over the transcriptional activating properties of MLL-AF4. These results lead to the conclusion that IRX1 is likely to be responsible for the t(4;11) ALL patient subgroup displaying the missing HOXA expression.

To validate previous observations with a pan-histone deacetylase inhibitor [26], we additionally performed experiments in the presence of 300 nM TSA. Ahmad *et al.* have recently shown that class I HDACi abrogates the dominant functions of MLL-AF4 on *ALOX5* gene transcription by the reactivation of endogenous MLL protein. As shown in Figure 2C, there are no effects of TSA on the transcription rate of *GAPDH*. Similarly, no significant changes can be observed regarding the expression of *IRX1* and *MLL-AF4*. However, when investigating *HOXA3-HOXA13* neither IRX1 nor MLL-AF4 is now able to suppress or activate *HOXA* genes. By contrast, all *HOXA* genes are transcribed equally high, indicating that the HDACi administration resulted in a functional block of MLL-AF4-derived properties. Based on these data and data obtained in our previous study [26], we conclude that HDACi acts dominant over IRX1 and concomitantly reactivates endogenous MLL which competes with the oncogenic MLL-AF4 (Figure 2E).

To understand the IRX1-mediated repression of *HOXA* genes in more detail, we performed immunoprecipitation (IP) experiments. MLL-AF4 was precipitated in the absence or presence of IRX1 (see Supplementary Figure S1: compare lanes 3 and 4). The precipitates were tested in Western blots for known “binding” and “non-binding” proteins. No changes could be seen for MEN1, LEDGF and DOT1L. In addition, factors known to be responsible for the transcriptional repression of wildtype MLL, like BMI-1 or CYP33, were absent. IRX1 was co-precipitated together with MLL-AF4, indicating that the observed repression of *HOXA* gene transcription is presumably due to a direct binding of IRX1 to the MLL-AF4 complex at target promotors. In the presence of IRX1 the composition and amount of the bound proteins was not changed, indicating that IRX1 binds to MLL-AF4 in a non-competitive manner.

**Figure 3:**
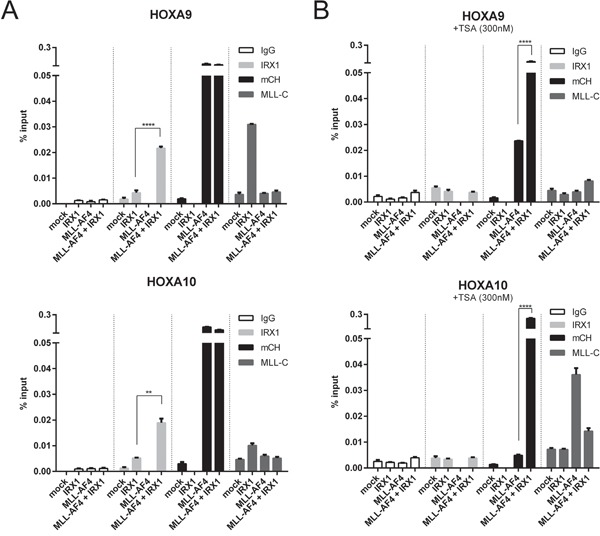
Chromatin-IP experiments to functionally investigate the IRX/MLL-AF4 interaction at MLL-AF4 target promotors Either mock-, *IRX1*-, *MLL-AF4*- or *IRX1/MLL-AF4*-transfected cell lines were investigated by ChIP. ChIP experiments were performed by using different antibodies (IgG, mCh (mCherry), MLL-C and IRX1). One representative experiment measured in triplicates is displayed. **A.** Experiments on the *HOXA* promotors without (left panels) or **B.** with 300 nM TSA (right panels). Binding of IRX1 became enhanced at these promotors when MLL-AF4 was present in the cell. Binding of MLL-AF4 to these promotors was very strong, independent of IRX1. Binding of wildtype MLL was enhanced in the presence of IRX1, but reduced in the presence of MLL-AF4. Upon TSA administration, binding of MLL-AF4 became reduced but retained in the presence of IRX1.

Next, we performed ChIP experiments with HEK293T cells expressing either IRX1, MLL-AF4 or both. We focussed on the *HOXA9* and *HOXA10* promotor regions to examine the functional consequences of an IRX1/MLL-AF4 complex formation (Figure 3A). A second set of experiments investigated the molecular effects of TSA on both *HOXA* promotors (Figure 3B).

IRX1 showed only a weak binding to both *HOXA* promotor regions. This binding was significantly increased in the presence of MLL-AF4 (Figure 3A; second panels, light grey bars). Binding of MLL-AF4 to both *HOXA* promotors was strong, irrespective whether IRX1 was present or not (third panel, black bars). In the absence of MLL-AF4, IRX1 enhanced the binding of wildtype MLL to both promotors (much stronger at the *HOXA9* promotor; fourth panel, dark grey bars). This may indicate the presence of a common docking site for IRX1 present in both multi-protein complexes.

When performing the same experiments in the presence of 300 nM TSA (Figure 3B), binding of MLL-AF4 to both promotors was reduced to different extends (weak at the *HOXA9* promoter, strong at the *HOXA10* promotor), while the recuitment of endogenous MLL was enhanced at the *HOXA10* promotor.

While IRX1 acts dominant over MLL-AF4 and functionally interfered with properties of the MLL-AF4 fusion protein at target site promotors, the addition of TSA blocked the functions of both proteins. This is identical to what has already been published for the MLL-AF4 fusion protein at the *ALOX5* promotor [26].

### Other IRX1 target genes

**Figure 4:**
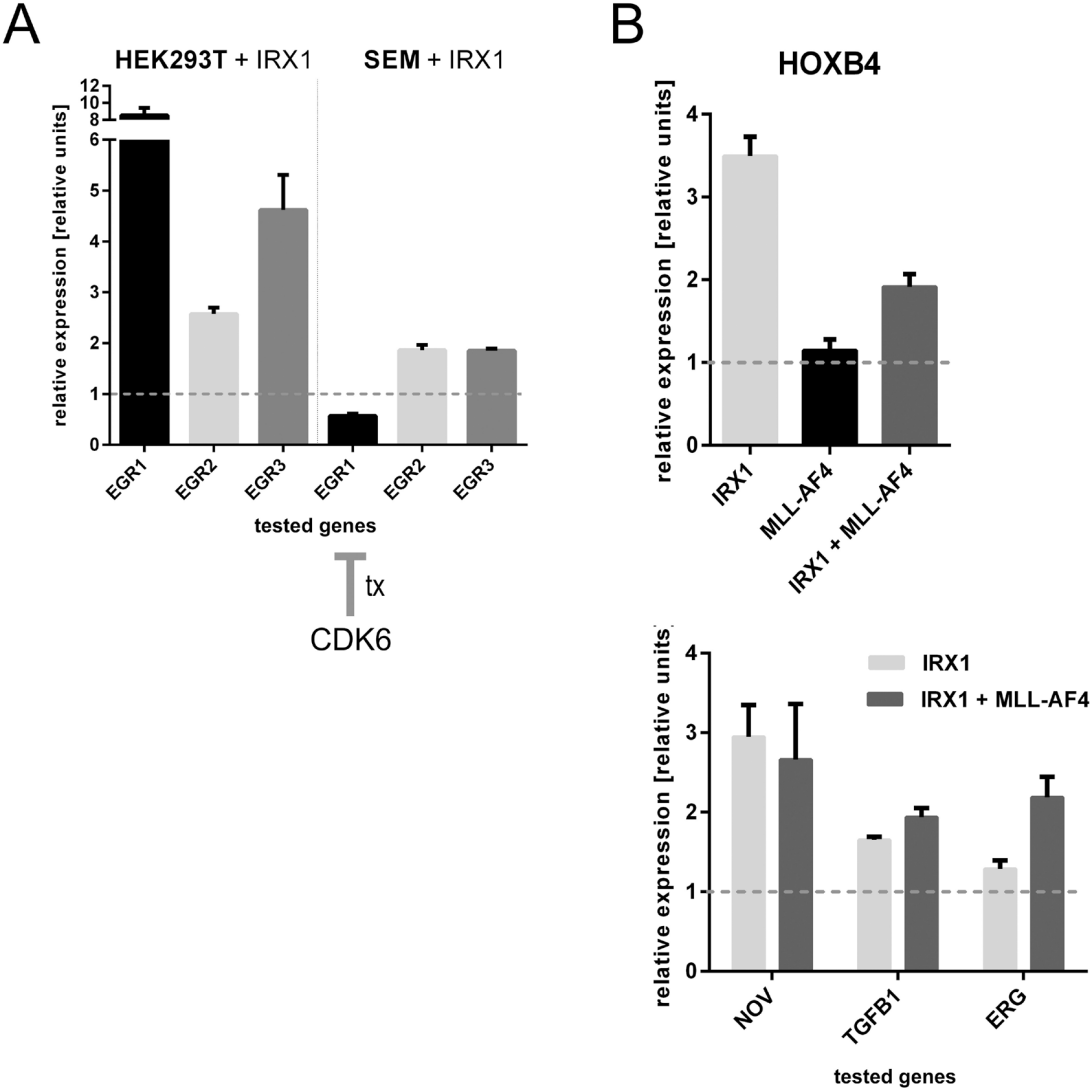
Consequences of IRX1 expression on the EGR1-3 and HOXB4 target genes **A.** Consequences of IRX1 overexpression in HEK293T and SEM cells regarding the *EGR1-3* target genes (n=3). While HEK293T cells strongly activate *EGR1* and *EGR3*, *EGR2* was only slightly enhanced in its transcriptional properties. SEM cells displayed the same effects besides *EGR1* expression. This is due to the high expression of CDK6. **B.** *HOXB4* was found to be upregulated upon IRX1 expression. *HOXB4* is not a target gene of MLL-AF4. Known downstream target genes of HOXB4, like *NOV*, *TGFB1* and *ERG*, were upregulated in the presence of IRX1 (n=3).

Besides *HOXA* genes, IRX1 activated a whole series of interesting target genes (GEO Acc. No. GSE75376). *EGR3* was one of the most upregulated target gene (Figure 1: 28-fold; p-value 0.00002). The EGR protein family is quite important for regulating a homeostatic process in hematopoietic stem cells (HSC). In particular, EGR proteins coordinate proliferation and migration of HSCs [27]. As shown in Figure 4A, the expression of IRX1 was able to induce transcription of *EGR1* (8-fold), *EGR2* (2.5-fold) and *EGR3* (4.5-fold). By contrast, the t(4;11) cell line SEM displayed only moderately activated (2-fold) *EGR2* and *EGR3*, because they overexpress CDK6 which is known to suppress transcription of the *EGR1* gene [28,29].

Next, we investigated the *HOXA* gene transcription after overexpression of EGR1-3. All three proteins decreased the transcription of several *HOXA* genes, affecting mostly *HOXA6* (Supplementary Figure S2A). However, the general effect was not as profound as seen in Figure 1A with IRX1. From these data we concluded that EGR expression is presumably more responsible to maintain hematopoietic stem cells. We also tested whether *EGR1-3* are direct targets of IRX1, however, we could not confirm this with ChIP experiments (data not shown).

### IRX1 directly activates the *HOXB4* gene

Another identified target gene is *HOXB4*. One of its downstream targets, *TGFB1*, was found on the high-score list of transcriptionally activated genes (Figure 1A;8-fold, p-value 0.00002). Since HOXB4 is important for the hematopoietic stem cell compartment, we investigated the influence of IRX1 and/or MLL-AF4 expression on *HOXB4* gene transcription.

As summarized in Figure 4B, *HOXB4* became transcriptionally activated (3.5-fold) when IRX1 expression was induced while no significant effect was detected in the presence of MLL-AF4. When both proteins were co-expressed, an increase in *HOXB4* gene transcription was still detected. Next, we tested some of the known downstream targets of HOXB4 (*NOV*, *TGFB1* and *ERG*). All three genes became transcriptionally activated when IRX1 expression was induced. This effect was not diminished in the presence of MLL-AF4 which indicated again that the activation of *HOXB4* is a genuine and novel function of IRX1.

Next, we analyzed whether *HOXB4* is a direct target gene of IRX1 by ChIP experiments. *HOXA9* and *MEIS1* were used as experimental controls. We analyzed the promotor region and the first intron as internal control. As shown in Supplementary Figure S2B, we were able to monitor a significant enrichment of IRX1 at the *HOXB4* promotor. We did not see any enrichment of IRX1 at the *HOXA9* promotor because this binding only occurs in the presence of MLL-AF4 (see Figure 3A). However, we obtained a minimal enrichment at the *MEIS1* promotor and first intron. *MEIS1* was transcriptionally suppressed upon IRX1 expression (Figure 1). From all these results we conclude that *HOXB4* is a *bona fide* target gene of IRX1 and that the transcriptional repression of *MEIS1* is presumably due to IRX1 binding.

### Confirmatory studies in the t(4;11) cell line SEM

Finally, we generated a transgenic SEM cell line expressing IRX1 upon doxycyline administration. For this purpose, we used our Sleeping Beauty system and the AMAXA Nucleofactor^®^ technology in combination with “puromycin pulse selection” to generate a stable SEM cell line over weeks. As shown in Supplementary Figure S3, transgenic SEM cells were expressing GFP from the Sleeping Beauty vector backbone (lower right panel) while normal SEM cells did not (only background fluorescence). *IRX1* gene transcription was induced by doxycycline administration as shown by the displayed Q-PCR and Western blot experiments. IRX1 expression in SEM cells caused a downregulation of MLL-AF4-driven *HOXA* gene transcription (*HOXA5*, *HOXA9* and *HOXA10*) while *HOXA7* was not affected in the presence of IRX1.

## DISCUSSION

High risk t(4;11) leukemia patients can be separated into the *HOXA^hi^*- and *HOXA^lo^*-subgroups [20]. This unusual finding is important because *HOXA^lo^*-patients display a dismal prognosis [21,22]. Within the signatures of up- and downregulated genes in these two cohorts, *IRX1* has been identified as the major upregulated gene in *HOXA^lo^*-patients. This provoked us to investigate this finding in more detail to understand the impact of ectopic expression of IRX1.

Here, we established a series of cell culture model systems where effects exerted by IRX1 could be investigated in an inducible fashion. We used the recently established Sleeping Beauty system [24] to express IRX1, MLL-AF4 or EGR1-3 proteins. We were able to confirm the initial observation made in t(4;11) patient cells, namely that IRX1 expression is correlated with the transcriptional downregulation of distinct *HOXA* genes (*A5, A9, A10*). Of note, we successfully used the Sleeping Beauty technology also to genetically modify the t(4;11) cell line SEM. So far, only lentiviral transduction allowed to genetically modify SEM cells, while any attempt to simply transfect t(4;11) cell lines was yet unsuccessful. This is the first time that a t(4;11) cell line could be genetically manipulated in a stable manner by using a non-viral gene transfer technology. In these modified SEM cells, IRX1 expression was able to downreglated the tested *HOXA* genes (Supplementary Figure S3B), validating again that IRX1 acts in a dominant-negative fashion over MLL-AF4.

IRX1 was able to decrease the transcriptional activity of several *HOXA* genes (Figure 2B). To our surprise *HOXA7* was not affected, although it is a known target gene of MLL fusions in hematopoietic and AML cells. However, *HOXA7* is usually not highly deregulated in biopsy material deriving from ALL patients (more *HOXA9* and *HOXA10*), suggesting that a deregulation of *HOXA7* is presumably depending on the investigated cell type or lineage.

IRX1 binds to the MLL-AF4 fusion protein complex without changing its composition (see Supplementary Figure S1). Subsequent ChIP experiments on *HOXA9* and *HOXA10* promotors revealed that binding of IRX1 occured only in the presence of MLL-AF4. This effect was diminished in the presence of the pan-HDAC inhibitor TSA. Ahmad and coworkers have recently shown that TSA administration results in a replacement of MLL-AF4 by endogenous MLL at the *ALOX5* promotor [26]. Here, we observed a similar result because the HDAC inhibitor TSA led to a strong reduction of bound MLL-AF4 at the tested *HOXA* target gene promotors (Figure 3), accompanied by an increased binding of endogenous MLL (see lane 3 with MLL-AF4). TSA administration alone was not able to enhance binding of endogenous MLL to this extend (see lanes 1 and 2 with mock or IRX1). This may indicate that binding of MLL-AF4 to its target genes may change the chromatin in a way that binding of endogenous MLL was enhanced to replace the MLL-AF4 oncoprotein when activated by TSA. Another explanation could be the interaction of MLL-AF4 with the endogenous AF4 complex which then confers enhanced H3K36_me2_ and H3K79_me2/3_ signatures. This typical “gene body signature” may be responsible for the observed enhancement of MLL binding. The concomitant presence of MLL-AF4 and IRX1, however, slightly reduced the binding efficiency of MLL when binding to these target promoters. A potential explanation could be that IRX1 blocks an interaction of MLL-AF4 with the endogenous AF4 complex. This could potentially explain the different binding properties of MLL-AF4 for known target gene promotors.

**Figure 5:**
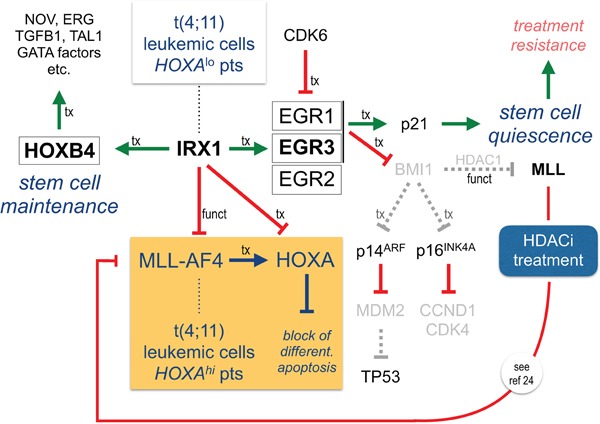
IRX1 functionally represses MLL-AF4 but transcriptionally activates HOXB4 and EGR genes IRX1 activates *EGR1*-3 (indirect) and *HOXB4* (direct). Since the IRX1 protein inhibits the transcriptional activator function of MLL-AF4, *HOXA* genes are repressed in the presence of IRX1. EGR1 and EGR3 are both able to activate p21. The tumor suppressor CDKN1A/p21 mediates quiescence of tumor stem cells. Concomitantly, a downregulation of BMI-1 causes cell cycle arrest and upregulation of TP53. HOXB4 drives a stem cell maintenance program. This stem cell like regulatory scheme is on top of the *HOXA* profile, known to be typical for *MLL-r* leukemia. The *HOXA* profile blocks differentiation and enhances proliferation and clonogenic growth. We assume that t(4;11) leukemic cells are enabled to switch between the “*HOXA*”- and the “*IRX1*”-driven programs. Most importantly, HDACi re-activates endogenous MLL that competes with oncogenic MLL-AF4 for binding at target promoters. This counteracts the oncogenic activities deriving from MLL-AF4 and results in physiological transcription rates (see also ref 26). HDACi also counteracts IRX1-mediated actions.

Noteworthy, IRX1 increased the transcription of several target genes well-known for their functional importance in the hematopoietic system. Among them, proteins of the *EGR* transcription factor family were identified. EGR1 controls the transcription of *CDKN1A* (p21) and subsequently the quiescence of hematopoietic stem and precursor cells [30–32]. EGR1 downregulates BMI-1 [33], a factor that controls the repressive state of MLL together with CYP33 [34–36]. BMI-1 inhibits the transcription of *CDKN2A* coding for p14^ARF^ [37,38] and p16^INK4A^ [39]. p14^ARF^ itself represses transcription of *MDM2* [40], and thus, enables the activation of TP53 [41], while p16^INK4A^ blocks the transcription of *CDK4* and *CyclinD1* [42,43] and therefore affects the cell cycle. All these interesting links from the literature have been implemented into a model that is summarized in Figure 5.

Based on the data presented here and already published data from many others, the MLL-AF4 fusion protein causes the activation of *HOXA* genes. This is associated with a differentiation arrest, increased proliferation and enhanced clonogenic growth. However, leukemic cells bearing a t(4;11) translocation are somehow enabled to activate the *IRX1* gene. IRX1 represses transcription of *HOXA*, *MEIS1* and *PBX1-3* genes. On the other hand, IRX1 activates *EGR2-3*, while EGR1 is repressed due to overexpressed CDK6 in t(4;11) cells (see Figure 4A) [28,29].

IRX1 activates *HOXB4* which is on the apex of an important stem cell program that allows the maintenance and expansion of stem cells [45–49]. It is highly likely that this mechanism is necessary to induce and maintain leukemic stem cells. This is most likely the reason why t(4;11) patients with IRX1 expression have a higher risk of relapses [21,22].

Another interesting aspect is the repression of MDM2 which causes an increase of available TP53. This may result in a higher fidelity of DNA repair processes. This is indeed one of the phenomenons of t(4;11) leukemia, because these cells do usually not show an accumulation of recurrent secondary mutations [44]. Finally, a downregulation of *Cyclin D1* and *CDK4* via p16^INK4A^ may even help to control the quiescent state and may result in therapy resistance.

About 6 years ago, *HOXA^hi^*- and *HOXA^lo^*-patients have been first described in pediatric t(4;11) leukemia patients [20]. Now, we provide a first rational for this finding and a molecular explanation for the clinical behavior of *HOXA^lo^*-patients. In addition, our experimental findings clearly indicate the importance of HDACi to functionally interfer with genetic programs induced by MLL-AF4. In addition, the adverse effects of IRX1 in terms of establishing a “stem cell like program” can also be diminsihed by HDACi administration. This very interesting observation has potential implications for the treatment of t(4;11) leukemia patients in the future.

## MATERIALS AND METHODS

### Cell culture, transfections

HEK293T cells were grown in DMEM with 10 % (v/v) FCS (GE Healthcare), 2 mM L-glutamine (GE Healthcare) and 1 % (v/v) Pen Strep (GE Healthcare) at 37°C and 5 % CO_2_. SEM cells were cultured under the same conditions with RPMI 1640 supplemented with 10 % (v/v) FCS and 1 % (v/v) Pen Strep (GE Healthcare). Transient and stable transfections of HEK293T cells were performed with polyethylenimine (PEI). The transfections of SEM cells were performed with the AMAXA^®^ Nucleofector^®^ (Kit R, program T-016, Lonza AG).

### Sleeping Beauty system

Cells were transfected with PEI or the AMAXA^®^Nucleofector^®^-Kit following the instructor's manual. For stable integration of all Sleeping Beauty vectors, all vector constructs were co-transfected with a 1/10 amount of the SB transposase vector SB100X [50]. Twenty-four to 48 h after transfection cells were treated either with 300 μg/ml hygromycin, 2 μg/ml puromycin or 8 μg/ml blasticidin. The selection was proceeded until all cells were carrying the fluorescent markers integrated into the genome. The expression of transgenes was always induced by adding 1 μg/ml doxycycline for 48 h to the culture medium. The following cell line models were established: HEK293T with inducible *IRX1*, with inducible *MLL-AF4-mCh* and with both inducible transgenes. In addition we also established HEK293T cells with inducible *EGR1*, *EGR2* and *EGR3*, as well as SEM cells with inducible *IRX1*.

### RNA extraction and cDNA synthesis

The total RNA of 5 × 10^6^ − 1 × 10^7^ cells was extracted by the RNeasy^®^ Mini Kit (Qiagen) after the induction of transgenes with doxycycline. One μg total RNA was subsequently reverse transcribed into cDNA by using the SuperScript^®^ II (Invitrogen).

### Real-time PCR analysis

All quantitative PCR analyses were performed with the StepOnePlus™ System (Applied Biosystems). All measurements were normalized to the Ct values of *GAPDH* of mock transfected cells (set to 1.0) and were analyzed in triplicates. The results were evaluated by the comparative ΔΔCt method. The results were shown as mean ± s.e.m. of at least three independent experiments.

The following primers were used for the Q-PCR experiments: GAPDHfor 5′-GGTCACCAGGGCTGCTTTTA-3′; GAPDHrev 5′-CGTTCTCAGCCTTGACGGTG-3′; HOXA3for 5′-GTGGCCAAACAAATCTTCCCC-3′; HOXA3rev 5′-CAGGTAGCGGTTGAAGTGGA-3′; HOXA4for 5′-AAGACCACAAACTGCCCAAC-3′; HOXA4rev 5′-GGTGTGGGCTCTGAGTTTGT-3′; HOXA5for 5′-CCGGACTACCAGTTGCATAAT-3′; HOXA5rev 5′-ATTGTAGCCGTAGCCGTACC-3′; HOXA6for 5′-AGTCTCCCGGACAAGACGTA-3′; HOXA6rev 5′-GCTGTCGGGTTTGTACTGCT-3′; HOXA7for 5′-TGAGGCCAATTTCCGCATCT-3′; HOXA7rev 5′-CGTCAGGTAGCGGTTGAAGT-3′; HOXA9for 5′-CCACGCTTGACACTCACACT-3′; HOXA9rev 5′-AGTTGGCTGCTGGGTTATTG-3′; HOXA10for 5′-CTTCCGAGAGCAGCAAAGCC-3′; HOXA10rev 5′-ACTCCTTCTCCAGCTCCAGT-3′; HOXA13for 5′-TGCCCAACGGCTGGAA-3′; HOXA13rev 5′-TAAGGCACGCGCTTCTTTCT-3′; HOXB4for 5′-CCCTGGATGCGCAAAGTT-3′; HOXB4rev 5′-AATTCCTTCTCCAGCTCCAAGA-3′; IRX1for 5′-GGATCTCAGCCTCTTCTCG-3′; IRX1rev 5′-GTGGAGACCTGCGTGAGG-3′; MLL-AF4for 5′-CCCAAAACCACTCCTAGTGAG-3′; MLL-AF4rev 5′-TTCACTGTCACTGTCCTCACTGTC-3′; EGR1for 5′-AGCCCTACGAGCACCTGA-3′; EGR1rev 5′-CTGACCAAGCTGAAGAGGGG-5′; EGR2for 5′-GGTTTTGTGCACCAGCTGTC-3′; EGR2rev 5′-TGGGAGATCCAACGACCTCT-3′; EGR3for 5′-ACAATCTGTACCCCGAGGAGA-3′; EGR3rev 5′-TCCCAAGTAGGTCACGGTCT-3′; NOVfor 5′-TGCTACTGCCTGAGCCTAAC-3′; NOVrev 5′- GTCCACTCTGTGGTCTGTTCA-3′; TGFB1for 5′- GTACCTGAACCCGTGTTGCT-3′; TGFB1rev 5′- GTTGCTGAGGTATCGCCAGG-3′; ERGfor 5′-GACGACTTCCAGAGGCTCAC-3′; ERGrev 5′- GCATGCATTAACCGTGGAGAG-3′.

### Chromatin immunoprecipitation

ChIP experiments were performed using the protocol of Abcam. Stably transfected cells (2 × 10^7^ cells on a 145-mm cell culture plate) were induced with doxycycline for 48 h. For the co-transfected samples stably transfected HEK293T cells, containing the *IRX1* transgene, were transiently transfected with *MLL-AF4-mCH*, and *vice versa*. 300 nM TSA or the same volume DMSO was added 16 h before harvest. For double fixation, the cells were incubated with 2 mM di(N-succinimidyl) glutarate for 45 minutes and 1 % (v/v) formaldehyde for 10 minutes. Sheared chromatin was incubated with magnetic A/G beads and the following antibodies overnight: normal goat IgG (sc-2028, Santa Cruz), IRX1 (ab72642, Abcam), MLL-C (61295, Active Motif), mCherry (ab167453, Abcam). For quantitative PCR analysis, the following primers were used: HOXA9prom.for 5′-ATGCTTGTGGTTCTCCTCCAGTTG-3′; HOXA9prom.rev 5′-CCGCCGCTCTCATTCTCAGC-3′; HOXA9intr.for 5′-AGTGGCGGCGTAAATCCT-3′; HOXA9intr.rev 5′-TGATCACGTCTGTGGCTTATTT GAA-3′; HOXA10prom.for 5′-CGCAACCACCCC AGCCAG-3′; HOXA10prom.rev 5′-TTGTCCGCCG AGTCGTAGAGG-3′; HOXB4prom.for 5′-TTAA ATATCCGGGGCCCCATC-3′; HOXB4prom.rev 5′-AAGTCCTTTTGGAAAAATTCAGTGG-3′; HOXB4intr.for 5′-AATCCGTATTTAAGCAGAGAGTTGA-3′; HOXB4intr.rev 5′-TTTGCTCACTTCTCCAGCCAA-3′; MEIS1prom.for 5′-CGGCGTTGATTCCCAATTTATTTCA-3′; MEIS1prom.rev 5′-CACACAAACGC AGGCAGTAG-3′; MEIS1intr.for 5′- TCTCAGCGC CTCCAAATCTTG-3′; MEIS1intr.rev 5′-TTTGTGTGTGTGAAATTTAGCTATTTAGGTTTT-3′.

The results obtained from the ChIP experiments are shown as mean ± s.e.m. of one representative experiment measured in triplicates (except Supplementary Figure 2B: measured in duplicates or triplicates, depending on the available amount of isolated material). To determine the significance between two groups the unpaired two-tailed Student's t-test was used. Different p values are described as followed: ** p < 0.01; **** p < 0.0001.

### Co-Immunoprecipitation and western blot

Three 145-mm culture plates of stably transfected HEK293T cells containing the empty vector or *IRX1* were transiently transfected with *MLL-AF4-mCH* and the expression of all transgenes was induced with doxycycline for 48 h. Cells were lysed (150 mM NaCl, 20 mM HEPES, 0.4 mM EDTA, 2 % (v/v) Triton X-100, 10 mM ATP, protease inhibitors) and protein concentrations were adjusted using BCA-Assay (Thermo Scientific). The precipitates were incubated with the following antibodies: normal goat IgG (sc-2028, Santa Cruz) and mCherry (ab167453, abcam) and complexed overnight with magnetic A/G beads. After three washing steps, the co-precipitated proteins were eluted with Laemmli-buffer and analyzed using 6 % and 12 % SDS-PAGE. The proteins were transferred to PVDF membranes using the semi-dry TransBlot^®^ Turbo^®^ transfer system (Bio Rad). For “high molecular weight proteins” a wet blot was performed at 30 V for 16 h (Blotting buffer: 10 mM CAPS, 15 % methanol, pH 11). The membranes were blocked with 5 % milk in TBS-T for one hour. For protein detection, the membranes were incubated overnight with the following antibodies: BMI-1 (36-40 kDa, ab38295, Abcam), CDK9 (43 kDa, sc-484, Santa Cruz), CYP33 (33 kDa, Abcam), DOT1L/KMT4 (171 kDa, ab64077, Abcam), IRX1 (50 kDa, ab72642, Abcam), LEDGF (60-68 kDa, ab49281, Abcam), mCherry (MLL-AF4-mCH, 260 kDa, ab167453, Abcam), MEN1 (68 kDa, ab2605, Abcam). Followed by one hour incubation with either goat anti rabbit IgG (H/L): HRP (5196-2504, Bio Rad), peroxidase labelled anti-mouse (NIF825, GE Healthcare) or VeriBlot for IP secondary antibody (HRP) (ab131366, Abcam). The proteins were detected with the Clarity™ Western ECL Substrate (Bio Rad) using the Molecular Imager Chemi DOC™ XRS+ (Bio Rad).

## SUPPLEMENTARY FIGURES


