# Supplementary material for: The IRX1/HOXA connection: insights into a novel t(4;11)- specific cancer mechanism
Source: Oncotarget. 2016 May 9;7(23):35341–52. doi: 10.18632/oncotarget.9241 (PMC5085233; doi:10.18632/oncotarget.9241)
Supplement: Supplementary file 1 [file oncotarget-07-35341-s001.pdf]

The IRX1/HOXA connection: insights into a novel t(4;11)-specific cancer mechanism

SUPPLEMENTARY FIGURES

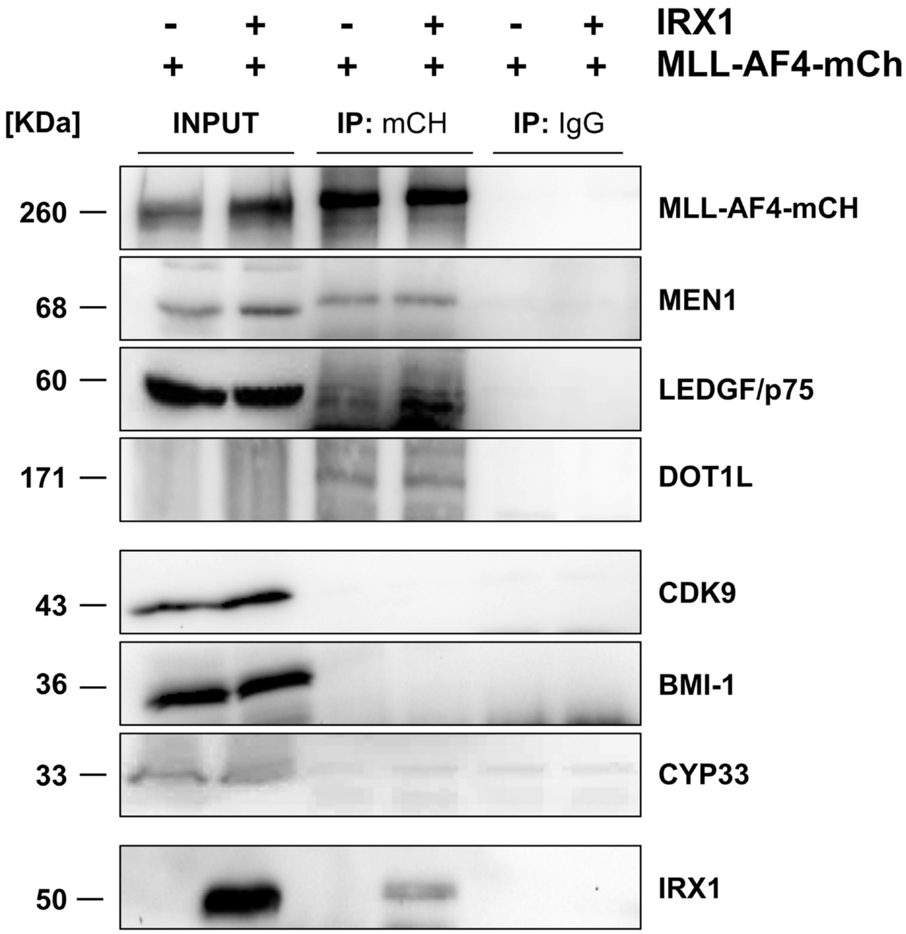

Supplementary Figure S1: Co-immunoprecipitation experiments of MLL-AF4. MLL-AF4 or MLL-AF4/IRX1 expressing cells were used to analyze the protein composition of the MLL-AF4 fusion protein. Input controls are shown on the left, unspecific IgG controls on the right. The MLL-AF4 fusion protein was precipitated by using antibodies against the C-terminal fused mCherry (mCh) protein. There was no change regarding the amount of co-bound MEN1, LEDGF/p75 or DOT1L. As expected, MLL-AF4 was negative for P-TEFb binding (measured by CDK9) or BMI-1 and CYP33. The IRX1 protein was precipitated with the MLL-AF4 fusion protein, demonstrating that IRX1 was able to bind to the MLL-AF4 complex.

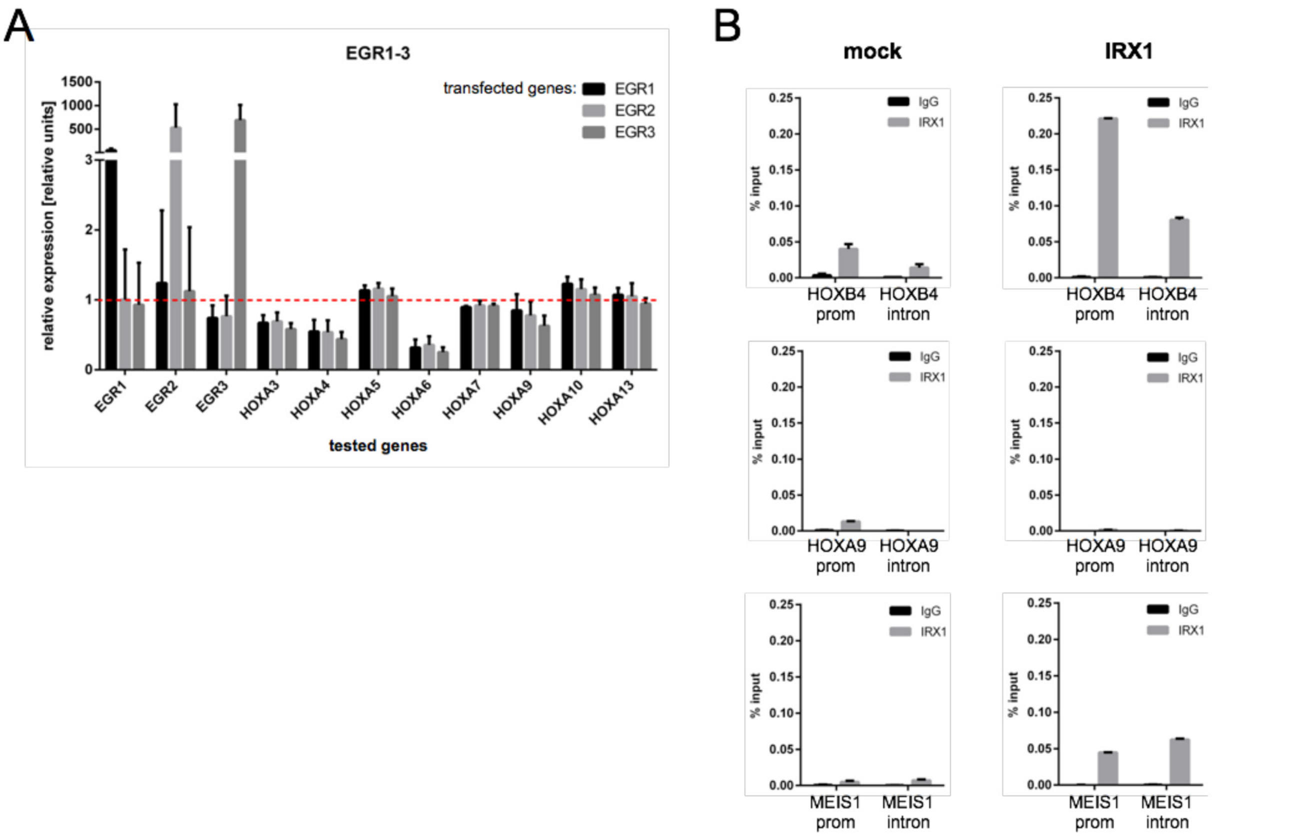

Supplementary Figure S2: Effects of EGR1-3 on *HOXA* gene transcription and identification of *HOXB4* as direct IRX1 target. A. Consequences of EGR1, EGR2 and EGR3 expression on the *HOXA* profile (n=3). Overexpression of all three EGR genes led to a slight downregulation of *HOXA3*, *HOXA4*, *HOXA6* and *HOXA9*. **B.** ChIP experiments on *HOXB4*, *HOXA9* an *MEIS1* promotors and first intron regions. One representative experiment measured in duplicates or triplicates is shown. As shown in the most right panels, IRX1 bound to the *HOXB4* promotor and the next intron. No IRX1 binding could be observed at the *HOXA9* promotor while a weak binding was observed at the MEIS1 promotor and within the first intron.

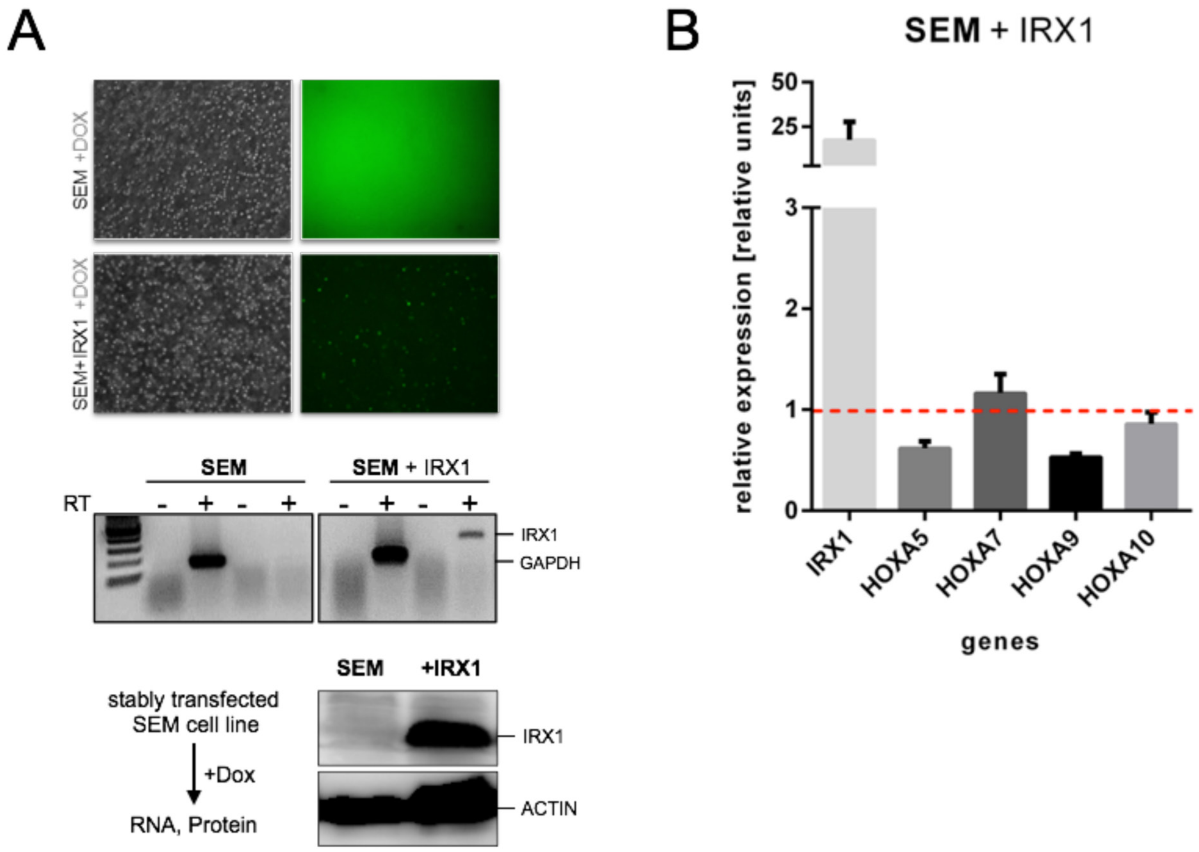

Supplementary Figure S3: Validation of the effects of IRX1 regarding the t(4;11) SEM cell line. A. SEM cells were stably transfected with the pSBtet::IRX1 construct constitutively expressing a GFP protein. *IRX1* transcripts and proteins were only visible in IRX1 expressing cells. **B.** The consequences of IRX1 overexpression in SEM cells led again to a downregulation of *HOXA5*, *HOXA9* and *HOXA10*, not affecting *HOXA7* (n=3) which is in line with the data presented in Figure 2.
